# Cyproheptadine inhibits in vitro and in vivo lung metastasis and drives metabolic rewiring

**DOI:** 10.1007/s11033-024-10033-6

**Published:** 2024-11-10

**Authors:** Ahmad Shannar, Md Shahid Sarwar, Parv Dushyant Dave, PoChung Jordan Chou, Rebecca Mary Peter, Jiawei Xu, Yuxin Pan, Fabio Rossi, Ah-Ng Kong

**Affiliations:** 1 Department of Pharmaceutics, Ernest Mario School of Pharmacy, Rutgers, The State University of New Jersey, 160 Frelinghuysen Road, Piscataway, NJ 08854 USA; 2 Graduate Program in Pharmaceutical Sciences, Ernest Mario School of Pharmacy, Rutgers, The State University of New Jersey, Piscataway, NJ 08854 USA; 3 Department of Medical Genetics, University of British Columbia, Vancouver, BC V6T 1Z1 Canada

**Keywords:** Lung cancer, Metastasis, Metabolomics, Syngeneic mouse model

## Abstract

**Background:**

Non-small cell lung cancer (NSCLC) accounts for 81% of lung cancer cases, among which over 47% presented with distant metastasis at the time of diagnosis. Despite the introduction of targeted therapy and immunotherapy, enhancing the survival rate and overcoming the development of resistance remain a big challenge. Thus, it is crucial to find potential new therapeutics and targets that can mitigate lung metastasis and investigate its effects on biomarkers, such as cellular metabolomics. In the current study, we investigated the role of cyproheptadine (CPH), an FDA-approved anti-histamine drug in lung metastasis in vitro and in vivo.

**Methods and results:**

CPH showed potent cytotoxicity on different lung cancer cell lines in vitro. Moreover, CPH decreased invasion and migration of LLC1 and A549 cells in Matrigel invasion transwell and plate scratch assays. The in vivo LLC1 syngeneic lung cancer model found decreased number of metastatic nodules on the surface of lungs of Setd7 KO mice compared to SETD7 WT. CPH treatment resulted in decreased growth of LLC1 subcutaneous tumors compared to untreated SETD7 WT. Finally, metabolomic study of tumor tissues showed rewiring of metabolomic pathways and downregulation of amino acids, such as arginine, serine, and glycine) in Setd7 KO and WT treated with CPH compared to untreated Setd7 WT mice.

**Conclusion:**

These findings identify CPH as a potential therapeutic agent to block metastasis in advanced NSCLC and suggest SETD7 as a potential target for the prevention of lung metastasis.

**Supplementary Information:**

The online version contains supplementary material available at 10.1007/s11033-024-10033-6.

## Introduction

According to the American Cancer Society, it is estimated that 238,340 people will be diagnosed with lung cancer, and 127,070 people will die from the disease in 2024 [1]. Non-small cell lung cancer (NSCLC) accounts for 81% of lung cancer cases, among which over 47% presented with distant metastasis at the time of diagnosis [2]. Recently, many studies have been conducted to uncover mechanisms of lung cancer. However, using traditional radiation and chemotherapy is still ineffective in curing metastatic lung cancer patients. The introduction of targeted therapy and immunotherapy helped prolong the survival duration. Despite that, the unsatisfactory survival and the development of resistance to these interventions remain major challenges [3]. Therefore, it is still necessary to further study the biomolecular mechanisms (e.g., cellular metabolome), explore potential biomarkers, and find new targets of lung cancer metastasis for the development of new therapeutic strategies.

Cyproheptadine (CPH) is an FDA-approved antihistamine drug that has been used clinically as an antiallergic and off-label use as an appetite stimulant and serotonin antagonist [4, 5]. Interestingly, CPH has been identified as potent Setd7 inhibitor with anti-tumor properties in various cancer types, including hepatocellular carcinoma, myeloma, leukemia, mantle cell lymphoma, breast cancer, and urothelial carcinoma by inducing apoptosis and cell cycle arrest [6–8], inhibiting histone deacetylase [9], estrogen receptor alpha [10], mTOR and β-catenin signaling pathways [11], and restoring epigenetic silencing of IRF6 [6–9, 11, 12]. The combination of CPH and thalidomide diminished hepatocellular carcinoma with lung metastasis [13] and CPH monotherapy caused remission of HCC patient with bone metastasis [14]. The synergistic effects of CPH in combination with other antineoplastic agents, such as bortezomib and sorafenib have also been reported in preclinical and clinical studies [9, 15]. However, detailed mechanism of action in lung cancer and lung cancer metastasis is yet to be explored. Hence, in this paper, our goal is to study CPH, a potential SETD7 inhibitor in the inhibition of lung tumorigenesis and metastasis.

Accumulating evidence suggests that the expression of Setd7 is associated with cancer development and progression [16]. Setd7 regulates cancer cell proliferation by influencing β-catenin stability in both in vitro and in vivo models [17]. Setd7 expression is associated with increased proliferation and poor prognosis of various cancers [18]. In our previous study, we observed that Setd7 knockdown decreased the colony formation in human prostate cancer PC-3 and LNCaP cells [19], and Ingenuity® Pathway Analysis of microarray data showed Setd7 KD attenuates cell growth/proliferative pathways and activates the cell apoptosis pathways [19, 20]. Setd7 has been identified as an oncogene in lung cancer [21]. A recent study shows Setd7 controls the proliferation and genotoxic drug resistance of lung cancer [22]. Several targets of Setd7 in lung cancer cells, including p53 and Mdm2, have been reported and knockdown of Setd7 suppressed the malignant properties of lung cancer cells [23–25]. Hence, Setd7 may play a crucial function in several physiological and pathological activities, such as cellular metabolism [26]. Amino acids, which are considered essential units for the survival of all cell types, experience reprogrammed metabolism in cancer. In this paper, we expand and focus on the role of Setd7 and its inhibitor in lung cancer metastasis via rewiring of cellular metabolism.

## Materials and methods

### Materials

Cyproheptadine (CPH) was purchased from Sigma-Aldrich, Inc. (St. Louis, MO, USA). Versene, trypsin–EDTA, fetal bovine serum (FBS), penicillin–streptomycin (10,000 U mL^−1^), puromycin, Dulbecco′s Modified Eagle′s Medium (DMEM) were purchased from Gibco Laboratories (Grand Island, NY, USA). Dimethyl sulfoxide (DMSO) was purchased from Sigma-Aldrich (St. Louis, MO, USA). The MTS reagent (3-(4,5-Dimethylthiazol-2-yl)-5-(3-Carboxymethoxyphenyl)-2-(4-Sulfophenyl)-2H-Tetrazolium) CellTiter 96 AQueous One Solution was obtained from Promega (Madison, WI, USA). Methanol (99%), and formic acid (98%) were purchased from Sigma-Aldrich (St. Louis, MO, USA). Acetonitrile and pure water were purchased from Honeywell Burdick & Jackson (Muskegon, MI, USA). Bicinchoninic acid (BCA) protein assay kit was purchased from Pierce Biotech (Rockford, IL, USA).

### In vitro studies

#### Cell lines culturing

Mouse Lewis Lung Carcinoma cell line (LLC1) was purchased from American Type Culture Collection (ATCC, catalog number CRL-1642) and cultured in high-glucose DMEM with 10% fetal bovine serum (FBS). Human lung cancer cell lines (A549 and H388) were cultured in DMEM with 10% FBS. Human bronchial epithelium normal cell line (Beas-2b) was cultured in Bronchial Epithelial Cell Growth Medium (BEGM) constituted with Bronchial Epithelial Cell Growth Basal Medium and Bronchial Epithelial Cell Growth Medium SingleQuots™ Supplements packs (Lonza, Walkersville, MD, USA). All cell lines were incubated at 37 °C in a humidified 5% CO_2_ atmosphere.

#### Cell viability assay

LLC1 and A549 cells were seeded in a 96-well plate at a density of 4 × 10^3^ cells per well overnight and then treated with either 0.1% DMSO as vehicle control or various concentrations of CPH (10–60 µM) for 24, 48, and 72 h in DMEM medium supplemented with 1% FBS. Cell viability was assayed using MTS reagents (3-(4,5-dimethylthiazol-2-yl)-5-(3-carboxymethoxyphenyl)-2-(4-sulfophenyl)-2H-tetrazolium) following the manufacturer's instructions.

#### Scratch migration assay

LLC1 and A549 cells were seeded overnight in 6 well plates with density of 1 × 10^6^ cells/well. Next, a scratch was generated by gliding the micropipette tip across the cell surface. The media was replaced with media containing CPH (20 and 30 µM) or 0.1% DMSO as control. The cells were incubated with the treatment for 24 h. Finally, the scratches were imaged by electron microscope. The average width of the scratch line was recorded on ImageJ. The width was normalized against the control. The assay was repeated three times, and the results were shown as mean ± SD.

#### Transwell Matrigel invasion assay

Cell invasion assays were performed in 24-well plates with 8 μm pore sized transwell chambers (Celltreat, catalog number 230639, Pepperell, MA). The upper compartment was coated with 10 ml Matrigel. A total of 2 × 10^4^ cells in low serum medium (0.1% BSA) were added to the upper chamber. The cells were serum-starved overnight in low serum medium (0.1% BSA). Then, cells were treated with CPH (20 and 30 µM) in a low serum medium (0.1% BSA) while full growth DMEM media (10% FBS) was added to the lower chamber. LLC1 and A549 cells were cultured for 24 h to observe migration. The migrated cells were fixed with 75% ethanol and stained with crystal violet for 15 min at room temperature. The crystal violet stain was then removed from the chambers, and cells were washed thrice with distilled water (dH2O). Cells on the upper chamber of the membrane were scraped off using a cotton swab. Cell migrations were captured by eluting the crystal violet stain with extraction buffer (2.5% methanol, 2.5% Isopropanol, 30% ethanol and 0.5% acetic acid) in dH_2_O and the eluted samples were transferred to a 96-well plate. The migrated stained cells were quantified by colorimetric measurement at 550 nm. The absorbance was measured using a microplate reader in accordance with the manufacturer’s instructions.

#### Protein lyses preparation and western blotting

LLC1, A549, H385, and Beas-2b cells (1 × 10^6^) were harvested using RIPA buffer supplemented with protease inhibitor cocktail (Sigma, St. Louis, MO, USA). Protein concentrations of each cleared lysates were measured using the BCA method (Pierce, Rockford, IL, USA). Total 45 µg proteins of each sample were separated by 4–15% SDS–polyacrylamide gel electrophoresis (Bio-Rad, Hercules, CA, USA). Then, the proteins were transferred to a polyvinylidene difluoride membrane (Millipore, Bedford, MA, USA) followed by blocking with 5% BSA in Tris-buffered saline-0.1% Tween 20 buffer. Then, the membrane was sequentially incubated with Setd7-specific primary antibodies and then HRP-conjugated secondary antibodies. Setd7 and GAPDH primary antibodies were purchased from Cell Signaling Technologies (Danvers, MA). The blots were visualized by SuperSignal enhanced chemiluminiscence detection system and recorded using a Gel Documentation 2000 system (Bio-Rad, Hercules, CA, USA). Finally, the bands were quantified using ImageJ software and normalized against Setd7 level in normal lung cell line Beas-2b cell.

### In vivo studies

#### Animal care and treatment

Setd7 knockout (KO) mice, originally described by Lehnertz et al. [27], were generously provided by Dr. Fabio M.V. Rossi from the University of British Columbia. Setd7 KO mice were backcrossed with C57BL/6J wild-type (WT) mice (The Jackson Laboratory, Bar Harbor, ME). To confirm the genotype from each animal, DNA was extracted by ear punching and analyzed by PCR using the following primers: 5′ geno forward primer, 5′-CCCTGAGCAGCCTTCTTTAATGGC-3′, 3′ geno forward primer, 5′-GGCTTGTGACACACAGCTCATTG-3′, and 3′ geno reverse primer, 5′-AGGCCTTCTCGGTTG ATGGACACCTT-3′. Setd7 KO and Setd7 WT mice exhibited one band at 400 and 450 bp, respectively. Male and female mice of Setd7 knockout of 8th generation or more were used in this study. 8 to 10-week-old mice were used and housed at Rutgers Animal Facility and maintained under 12-h light/dark cycles. All animals were allowed water and food ad libitum. All animal use procedures were in accordance with the NIH (National Institutes of Health) Guide for the Care and Use of Laboratory Animals and were approved by the Rutgers Institutional Animal Care and Use Committee.

#### Syngeneic lung cancer model development and treatment

100 μL serum-free media containing 1.5 × 10^5^ LLC1 cells was injected subcutaneously (s.c.) into the lower right quadrant of the abdomen of Setd7 WT and KO mice. WT mice were randomized into model control group and CPH treatment group. All groups have 8 mice (n = 8, 4 males and 4 females). Tumor volume was calculated as (length × width^2^)/2). When the tumor volume reached 100 mm^3^, the treatment group received intraperitoneal (i.p.) injection of CPH (20 mg/kg, three times a week until sacrifice) dissolved in 2% ethanol in PBS as a vehicle, where WT model control received the vehicle only. After 28 days of LLC1 cell injection, the mice were sacrificed, and the tumors and lungs were isolated. Tumor tissues were weighed before snap-freeze for RNA and metabolites extraction. The number of lung metastases was determined by counting the number of metastatic nodules on the lung surface under the microscope. Then half of the lung was fixed with formalin and embedded in paraffin for H&E staining. The other half was snap-frozen for further investigations.

#### H&E histological examination of lung tissues

Lung samples were inflated with phosphate buffer and placed in 10% formalin at room temperature for 24–48 h. The samples were then dehydrated in increasing concentrations (80, 95, and 100%) of ethanol, cleared in xylene, and embedded in Paraplast Plus (Fisher Scientific, Pittsburgh, PA, USA) using an automated platform (Leica). H&E staining was conducted as previously described [28]. Briefly, 3–5 sections of 5-micron thickness were prepared using motorized-rotary microtome. Sections were deparaffinized using xylene and then rehydrated with gradient water in alcohol solutions before being stained with H&E. The stained sections were examined and imaged under a light microscope (Nikon Eclipse E600, Japan).

#### LC–MS metabolomic analysis

LC–MS metabolomic analysis was performed in Metabolomics Shared Resources, Rutgers Cancer Institute of New Jersey (CINJ) as previously reported [28–30]. Metabolites were extracted from tumor tissues with 1 mL cold 40:40:20 methanol:acetonitrile:water solution with 0.5% formic acid, followed by 5 min incubation on ice and sequentially neutralized with 50 µL 15% NH_4_HCO_3_. The cleared supernatant was then used for LC–MS analysis.

LC separation was performed on a XBridge BEH Amide column (2.1 mm × 150 mm, 2.5 µm particle size, 130 Å pore size; Waters) coupled with a Waters XBridge BEH XP Vanguard cartridge (2.1 mm × 5 mm, 2.5 µm particle size, 130 Å pore size) guard column. The solvent A prepared by water/acetonitrile (95:5, v/v) with 20 mM NH3AC and 20 mM NH_3_OH at pH 9; and solvent B prepared by acetonitrile/water (80:20, v/v) with 20 mM NH_3_AC and 20 mM NH_3_OH at pH 9 in the following solvent B percentages over time: 0 min, 100%; 3 min, 100%; 3.2 min, 90%; 6.2 min, 90%; 6.5 min, 80%; 10.5 min, 80%; 10.7 min, 70%; 13.5 min, 70%; 13.7 min, 45%; 16 min, 45%; 16.5 min, 100%. The flow rate was set to 300 µL min^−1^ with an injection volume 5 µL. The column temperature was set at 25 °C. MS scans were obtained in positive ion mode with a resolution of 70,000 at *m*/*z* 200, in addition to an automatic gain control target of 3 × 10^6^ and *m*/*z* scan range of 72–1000.

Metabolomic pathways analysis was performed within the Web-based inference software MetaboAnalyst 5.0 (https://www.metaboanalyst.ca/).

#### Quantitative real-time polymerase chain reaction (RT-PCR)

Tumor tissue samples were homogenized and mRNA was extracted using mRNA extraction kit from (Thermo Fisher Scientific). The first-strand cDNA was synthesized from 1 µg extracted RNA using SuperScript III First–Strand cDNA Synthesis System (Invitrogen, Grand Island, NY, USA). To determine mRNA expression of specific genes, the cDNA was used as the template for real time PCR using Power SYBR Green PCR Master Mix (Applied Biosystem, Carlsbads, CA, USA). The primers were designed and ordered from Integrated DNA Technologies (IDT, Coralville, IA, USA) (Table S1, supplementary material). The mRNA expression was calculated as the fold change with normalization to the expression of glyceraldehyde 3-phosphate dehydrogenase (GAPDH) using the 2^−ΔΔCT^ method while GAPDH was used as an internal loading control.

#### Statistical analysis

Data are expressed as mean ± SD. Student’s *t*-test was used to evaluate the statistical significance of the results. *P* values less than 0.05 were considered statistically significant.

## Results

### CPH suppresses the growth of lung *cancer* cell lines in vitro

**Fig. 1:**
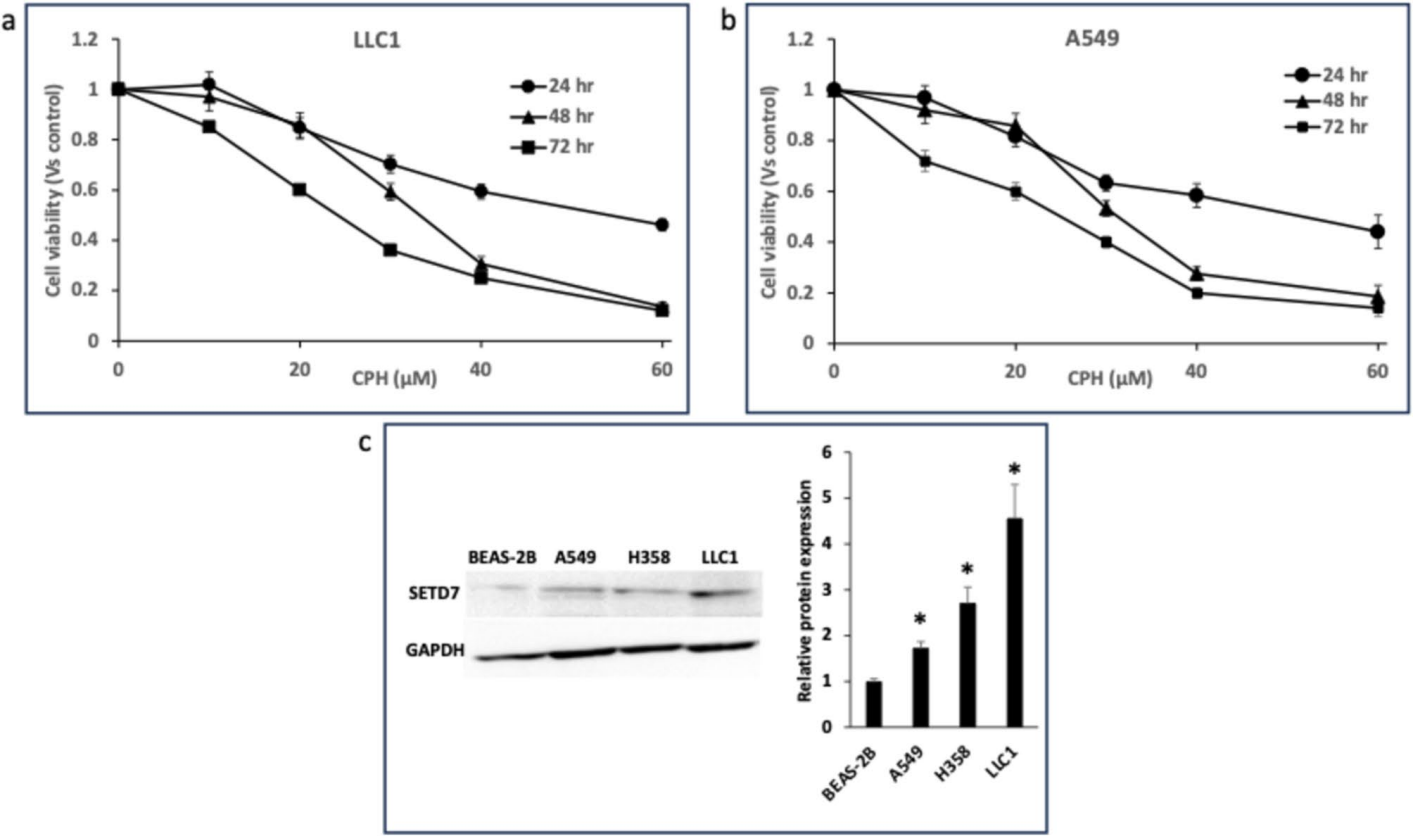
CPH-mediated cytotoxicity and regulation of Setd7-target genes in different lung cancer cell lines. **a** Cell viability of LLC1 cells after various concentrations of CPH treatment for 24-, 48- and 72-h; **b** Cell viability of the A549 cells after various concentrations of CPH treatment for 24-, 48- and 72-h. Cytotoxicity was determined by the MTS assay; **c** SETD7 protein expression in different lung cell lines quantified by Western Blot. The protein expressions were shown as fold expression compared to the normal lung cell lines Beas-2b. GAPDH was used as the housekeeping protein for normalization. All data are presented as the means ± SD of three independent experiments. *P < 0.05 indicates significant differences between the treatment groups and the control group. The student’s t-test was used to calculate the significance of the differences compared with the control

Treatment with CPH showed a time- and dose-dependent effect on the lung cancer cell lines viability (Fig. 1a, b). 20 µM CPH showed 15, 15, and 40% growth inhibition after treatment of LLC1 cells for 24, 48, and 72 h, respectively. While for A549, 20 µM CPH showed growth inhibition of 18, 14, and 40% after treatment for 24, 48, and 72 h, respectively. Higher concentration of CPH (30 µM) showed higher growth inhibition with 30, 41, and 64% for LLC1 and 37, 47, and 60% for A549 after treatment for 24, 48, and 72 h, respectively. These two concentrations were close to the IC_25_ (20 µM) and IC_50_ (30 µM), hence, they were selected for further in vitro studies.

### Setd7 is upregulated in advanced metastatic lung cancer cell lines

We performed WB to assess SETD7 protein expression in the different lung cancer cell lines with different metastatic characteristics. Compared to Beas-2b, which is considered a normal lung cell line, the advanced metastatic LLC1, H358 and A549 cells showed significantly upregulated protein expression of SETD7 (Fig. 1c).

### CPH attenuates invasion and migration of LLC1 and A549 cells in vitro

**Fig. 2:**
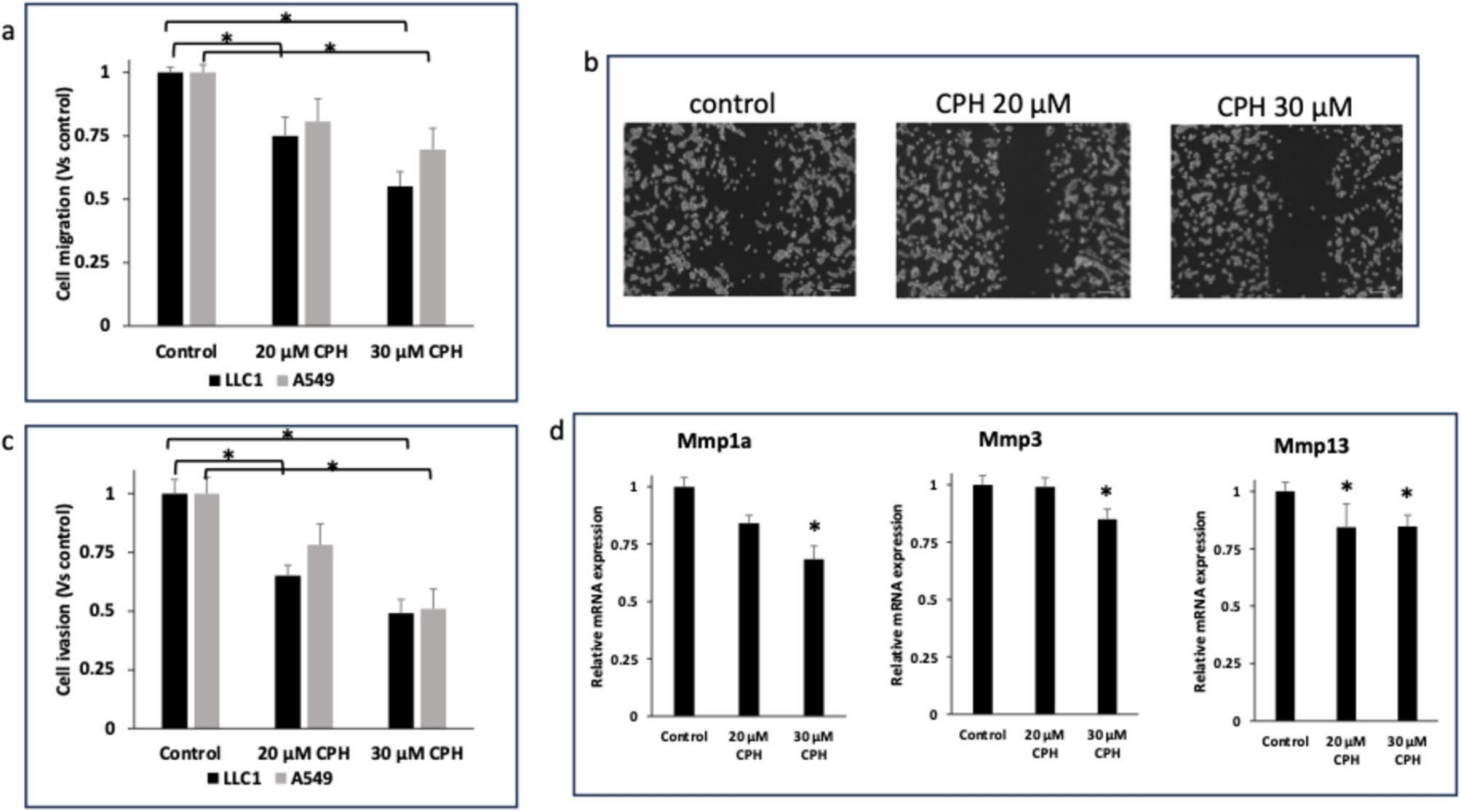
CPH inhibits cell migration and invasion and down-regulates MMPs gene expression in LLC1 and A549 cell lines. **a** Cell migration of LLC1 and A549 cells treated with different concentrations of CPH for 24 h using plate scratch assay (n = 3); **b** a representative image of plate scratch assay for LLC1 cells treated with different concentrations of CPH for 24 h. The average width of the scratch line was calculated with Image-J; **c** Transwell Matrigel cell invasion assay of serum-starved LLC1 and A549 cells after treatment of different concentrations of CPH for 24 h. Cell invasion was calculated by fluorescence plate reader of the eluted cells from the membrane and normalized against the control (0.1% DMSO). The assay was repeated 3 times (n = 3) and the mean ± SD; **d** MMPs mRNA expression in LLC1 cells treated with different concentrations of CPH for 24 h measured by RT-PCR. mRNA expressions were shown as fold change compared to the control group (0.1% DMSO). All data are presented as the means ± SD of three independent experiments. *P < 0.05 indicates significant differences between the treatment groups and the control group. The student’s t-test was used to calculate the significance of the differences compared with the control

The effect of CPH on migration was examined by plate scratch assay. Figure 2a, b show a dose-dependent inhibition of cell migration for CPH. 30 μM CPH inhibited 45 and 30% of total cell migration of LLC1 and A549 cells after treatment for 24 h, respectively. Although LLC1 cells show a higher migration characteristic (Fig. 2b, left panel), CPH treatment successfully decreased migration in dose-dependent manner (Fig. 2b, middle and right panels). Furthermore, cell invasion assay, examined using Matrigel invasion transwell, illustrated a significant inhibition effect of CPH on cell invasion potential of LLC1 and A549. Then, we investigated the matrix metalloproteinase (MMP), one of the most important metastasis pathways by assaying mRNA expression of several MMPs. The results show CPH significantly reduced the mRNA levels of MMP1, MMP9, and MMP13 (Fig. 2d).

In summary, the in vitro findings showed promising potential benefits of CPH as Setd7 inhibitor. CPH showed potential in inhibiting cancer cells growth and slowing metastasis by inhibiting cell migration and invasion. Hence, we decided to move forward and test CPH efficacy in vivo with syngeneic lung cancer murine model using subcutaneous injection of LLC1 cells in Setd7 WT and KO.

### Setd7 KO and CPH inhibit tumor growth and lung metastasis in vivo

We performed a syngeneic murine model to confirm the in vitro cytotoxicity, gene expression and cell migration/invasion results. Animal models can give a more accurate prediction of efficacy and can help answer the main question in this paper which is whether knocking out or inhibition of Setd7 can regulate lung metastasis.

The syngeneic murine model was established by injecting LLC1 cells s.c. in the lower right abdomen area. The subcutaneous inoculation of cancer cells enabled us to investigate the cytotoxic efficacy of CPH on the growth of tumors at the inoculation site and to assess the role of Setd7 in regulating metastasis to the lungs. LLC1 cells were injected into Setd7 WT and KO C57BL/6J. Then, WT mice baring LLC1 s.c. tumor were further divided into two groups: WT model control and WT treatment with CPH.

**Fig. 3:**
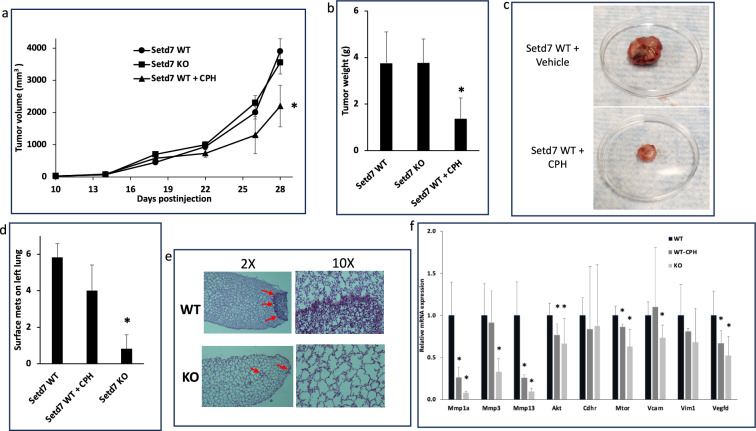
Setd7 KO and CPH regulate tumor growth and metastasis in the lungs in vivo. **a** Exponential tumor growth developed by LLC1 cells s.c. injection in Setd7 KO and Setd7 WT ± CPH presented as mm^3^ volume measured by caliper. Tumor volume was calculated following the formula: ½ × length × (width)^2^ (n = 8); **b** Tumor weight at the end of the study (n = 8); **c** Representative images for tumors of Setd7 WT mice treated with CPH vs vehicle; **d** Number of surface metastatic nodules on left lung counted under microscope (n = 8); **e** Representative images of H&E stained lung tissues of Setd7 WT and KO mice under electron microscope with 2× and 10× magnification power. Setd7 WT lung tissues show upregulated cell growth and large tumor/metastatic nodules (red arrows) compared to Setd7 KO lung tissues; **f** mRNA expression of metastasis-related genes for tumor tissues of Setd7 WT (control group), Setd7 KO, and Setd7 WT treated with CPH. mRNA expression was measured by RT-PCR and shown as fold change compared to Setd7 WT (control group). mRNA expression was measured for three samples per group (n = 3), and the mean ± SD were plotted. (Color figure online)

First, we investigated the efficacy of Setd7 inhibitor CPH on the growth of the LLC1 primary tumor in Setd7 WT mice. Mice in the treatment group received 20 mg/kg CPH i.p. three times a week until the mice were sacrificed. The treatment was initiated once the tumor size reached 100 mm^3^ (day 14 after LLC1 injection). The treatment dose of CPH was selected based on previously published papers [8, 10]. Tumor size was measured using Caliper twice a week. CPH significantly lowered the growth of the LLC1 tumor as shown in the growth rate graph (Fig. 3a). The mice were sacrificed on day 28 and the tumors were collected, weighed, and snap frozen for further analysis. The weight of tumors in mice treated with CPH was significantly lower (Fig. 3b, c).

Second, we evaluated whether knocking out Setd7 affects lung metastasis. While no difference in the primary tumor growth between Setd7 KO and WT mice was observed, we observed reduced metastasis in Setd7 KO mice compared to WT (Fig. 3d, e). To assess the effect of Setd7 on lung metastasis, we counted the number of surface metastatic foci under the microscope. We observed a significant reduction of small and large metastatic clusters (Fig. 3d). These findings indicate that the metastatic outgrowth in Setd7 KO mice has been greatly hampered. These results were confirmed by H&E staining of lung tissues (Fig. 3e), where the metastatic nodules are bigger in Setd7 WT compared to KO.

Finally, we ran qPCR to investigate mRNA expression of metastatic and apoptotic genes that could have been affected by Setd7 knocking out or inhibition by CPH in primary tumor. The mRNA expression levels of the metastasis-related genes and pathways were assessed. Mmp1a, Mmp3, and Mmp13, which engage in metastasis through facilitating invasion of tumor cells, were downregulated in Setd7 KO and CPH groups compared to Setd7 WT group. Also, Vegfd mRNA expression was shown to be decreased in Setd7 KO and CPH groups. Other metastasis and apoptosis-related genes such as mTOR and Akt were downregulated as well. The percent reduction of mRNA expression of all these genes was always higher in the Setd7 KO group compared to the CPH treated group.

### Setd7 KO and CPH treatment drive cellular metabolomic rewiring in tumor tissues

To unravel the potential underlying molecular links between Setd7 regulation and metastasis as well as the impact of CPH on metastasis through regulating cellular metabolic pathways and metabolites, the tumor tissue samples collected from Setd7 WT (model control), Setd7 KO, and Setd7 WT treated with CPH were assayed using LC/MS/MS to perform the metabolomic analysis. A total of 113 metabolites were identified. Among these identified metabolites, we found 19 and 24 metabolites were significantly regulated in Setd7 KO and CPH groups compared to Setd7 WT, respectively.

**Fig. 4:**
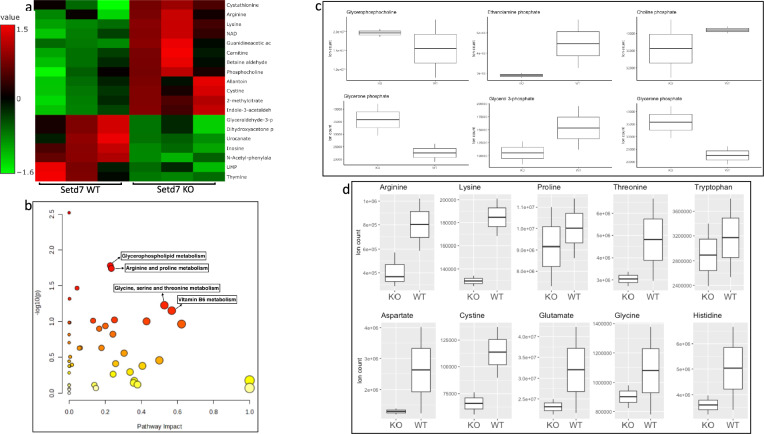
Setd7 KO drives metabolomic rewiring in tumor tissues. **a** Heatmap showing the top regulated metabolites in tumor tissues of Setd7 KO vs WT; **b** Metabolism pathway analysis; **c** Metabolites of glycerophospholipid metabolism pathway; **d** Quantified amino acids level in tumor tissues. All metabolites were assayed using LC/MS/MS of three replicates (n = 3). The metabolomic study was analyzed using Metaboanalyst 5 software

The top regulated metabolites in the Setd7 KO group compared with Setd7 WT were plotted in the heatmap (Fig. 4a). Pathway analysis from Setd7 KO vs. WT comparison group revealed glycerophospholipid metabolism and amino acid metabolism pathways among the top regulated (Fig. 4b). The glycerophospholipid metabolism pathway was further analyzed, and its metabolites were plotted in Fig. 4c. Setd7 KO group shows downregulation in amino acids levels (i.e., arginine, proline, glycine, serine, and threonine) compared to Setd7 WT (Fig. 4d).

**Fig. 5:**
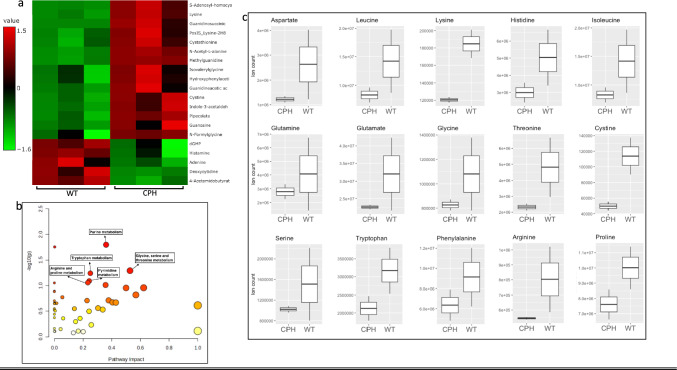
Setd7 inhibitor CPH drives metabolomic rewiring in tumor tissues. **a** Heatmap showing the top regulated metabolites in tumor tissues of Setd7 WT treated with vehicle and CPH; **b** Metabolism pathway analysis; **c** Quantified amino acids level in tumor tissues. All metabolites were assayed using LC/MS/MS of three replicates (n = 3). The metabolomic study was analyzed using Metaboanalyst 5 software

Comparison of CPH vs. WT control shows 24 significantly regulated metabolites (Fig. 5a). Pathway analysis from this comparison group revealed that the top-regulated metabolic pathways are related to amino acid metabolism (Fig. 5b). Hence, we next analyzed amino acid levels in CPH against WT control and found most of the amino acids were decreased in CPH group (Fig. 5c) which include arginine, proline, tryptophan, glycine, serine, and threonine.

## Discussion

Setd7 expression is associated with increased proliferation and poor prognosis of various cancers [18]. Previous studies reported that Setd7 promotes multiple malignant processes in breast cancer development [31]. Our previous study, we observed that Setd7 knockdown decreased colony formation in human prostate cancer PC-3 and LNCaP cells [19]. Nonetheless, SETD7 regulates cancer-related processes in a tissue type- and context-dependent manner [16]. This complexity makes interpreting results challenging. In lung cancer, SETD7 can inhibit cell proliferation by downregulating cyclins (CCNA1 and CCND1) [24]. SETD7 impacts metastasis by regulating genes such as Sonic Hedgehog pathway mediated by Gli3 methylation [32]. Furthermore, SETD7 methylation of HIF-1α inhibits angiogenesis and tumor growth [33].

On the other hand, SETD7-mediated methylation of KRAS leads to KRAS degradation and attenuation of the RAS/MEK/ERK signaling cascade [34]. Generally, SETD7 impact on cancer is multifaceted, involving diverse processes. Its context-dependent function highlights the need for further research to fully understand its potential diagnostic and therapeutic implications [16].

CPH has been identified as a SETD7 antagonist [10, 35, 36]. The binding kinetics of CPH to Setd7 were evaluated using a fluorogenic substrate-based assay, revealing its role as a competitive antagonist to the peptide substrate. This finding was further validated by X-ray crystallographic analysis. Notably, CPH exhibited selective antagonistic effects on Setd7, but not other histone methyltransferases (HMTs) [37]. CPH has been shown to possess anti-tumor properties in various cancer types, including hepatocellular carcinoma, myeloma, leukemia, mantle cell lymphoma, breast cancer, and urothelial carcinoma by inducing apoptosis and cell cycle arrest [6–8]. Some of these effects could be attributed to CPH’s ability to regulate essential proliferation pathways such as mTOR/β-catenin [11], p38/MAPK [38], and PI3K/AKT [7]. Yet, the detailed mechanism of action of Setd7 and its inhibitor CPH in lung cancer has not been fully explored. So, in the current study, we performed metabolomic analysis using an in vivo lung cancer tumor model established by LLC1 cells.

We first investigated the expression of Setd7 in different lung cell lines. WB results showed overexpression of Setd7 protein in advanced lung cancer cell lines such as A549, H358, and LLC1 compared to the normal lung cell line Beas-2b (Fig. 1). CPH has been recognized as a potent Setd7 inhibitor with anti-tumor activity in several cancer models [10, 39]. CPH showed dose- and time-dependent growth inhibitory effects on human (A549) and mouse (LLC1) lung cancer cells (Fig. 1).

Next, we focused on the effects of CPH on cell migration and invasion as indicators of the metastatic potential of advanced lung cancer cells. Scratch migration and transwell invasion assays show dose-dependent inhibitory effects of CPH on LLC1 and A549 cells (Fig. 2). MMPs are recruited by the cancer cells in the process of angiogenesis to break down the extracellular matrix and facilitate the formation of new blood vessels [40]. Our in vitro results show a downregulation of mRNA expression of several MMPs such as Mmp1a, Mmp3, and Mmp13 (Fig. 2). mRNA levels of MMPs and other metastasis-related genes were further investigated in the in vivo LLC1 syngeneic lung cancer model as discussed next.

To better understand lung metastasis and to confirm the in vitro results of CPH on lung cancer cell lines, we performed a syngeneic mouse lung cancer model. We established the model by injecting LLC1 cells s.c. which enabled us to not only study the CPH efficacy in inhibition of lung cancer cells growth but also to assess the role of Setd7 in lung metastasis. Furthermore, we utilized metabolomic analysis of in vivo tumor tissues to explore the intricate relationship between Setd7 and its inhibitor CPH with lung metastasis. Both Setd7 WT and KO mice were used for this study and Setd7 WT were further randomized to model control (received vehicle only) and treatment group (received 20 mg/kg CPH). Although Setd7 KO mice did not show a difference in primary tumor growth and size, metastatic nodules on lung surface were significantly decreased compared to Setd7 WT group (Fig. 3). On the other hand, CPH-treated mice showed a significant decrease in primary tumor size and volume. Setd7 has been identified as an oncogene in lung cancer [21]. In a previous study, we observed that Setd7 knockdown decreased colony formation in human prostate cancer PC-3 and LNCaP cells [19]. Previously, CPH diminished hepatocellular carcinoma with lung metastasis when combined with thalidomide [13]. Also, CPH monotherapy caused remission of HCC patients with bone metastasis [14]. It was reported that CPH interferes with mTOR and β-catenin signaling pathways to exert its anti-tumor effects [11]. Our analysis of in vivo tumor tissues by RT-qPCR showed Setd7 KO and CPH-treated mice showed attenuated apoptotic and metastatic pathways such as Akt/mTOR and MMPs (Fig. 3). Angiogenesis metastatic pathway gene expression (Mmp1a, Mmp3, Mmp13, and Vegfd) was decreased in Setd7 KO and CPH-treated mice. This inhibition was higher in Setd7 KO mice compared to CPH-treated mice. These findings show the significant role of Setd7 in lung metastasis in regulating different cellular signaling pathways. Moreover, knocking out Setd7 reduced lung nodules significantly but could not affect the primary tumor growth, which suggests that the presence of Setd7 in the environment might have a role in regulating tumor cells at the molecular level. Cellular metabolomic analysis illustrated the regulation of metabolite levels and the overall rewiring of cellular metabolomics in Setd7 KO and CPH-treated groups compared to control.

While the specific connection between SETD7 and cellular metabolomics remains an active area of research, understanding its role in these processes could shed light on novel therapeutic strategies for cancer and other diseases. The balance between stimulating or inhibiting SETD7 activity may hold promise in modulating cellular metabolism and its implications for health and disease [41]. To further investigate how Setd7 KO and its inhibitor CPH regulate metastasis at the molecular level and explore the related biomarkers, we analyzed tumor tissues’ cellular metabolites using LC/MS/MS technology. First, the pathway analysis was performed to gain a deeper understanding of the metabolic changes that occur when SETD7 is knocked out or inhibited by CPH. This analysis revealed significant alterations in glycerophospholipid metabolism and amino acid metabolism pathways, which are crucial for various cellular functions (Figs. 4 and 5). Most of the amino acids show a downregulation pattern in Setd7 KO and CPH-treated tumors. Amino acids play an essential role in tumorigenesis and metastasis as they serve as the building blocks for growing tumors [42]. Elevated serine biosynthesis has been observed in cancer cells associated with lung metastasis [43, 44]. Hence, some novel therapies target serine by decreasing its uptake and inhibiting its biosynthesis [37]. Arginine is another amino acid that was found to be downregulated in Setd7 KO and CPH mice (Figs. 4 and 5). Arginine depletion has shown better clinical success and enhanced antitumor efficacy of doxorubicin in several cancer types [45–47].

In summary, we found many similarities between CPH and Setd7 KO groups in terms of metabolomics and gene expression. However, further research is required to confidently attribute all the effects of CPH solely to SETD7 activity. While that was beyond the scope of this research paper, we recommend further investigation of CPH function using CRSPR technology to generate Setd7 KO cell lines. Studying the anti-cancer effects of CPH in SETD7 KO in vitro and in vivo models may help unravel the correlation between SETD7 inhibition and CPH anti-cancer effects. Furthermore, the interaction between CPH with prominent lung cancer pathways such as KRAS and EGFR and their inhibitors should be further evaluated in lung cancer cell lines that show different sensitivity to KRAS inhibitors.

## Conclusion

Advanced lung cancer with metastasis remains a challenging disease despite the recent advancement in targeted and immunological treatments. In our study, we show a potential new target for the treatment of lung metastasis. Setd7 inhibitors, such as CPH, may be a promising candidate for targeting the metabolomic rewiring of lung cancer cells. CPH showed efficacy in attenuating lung cancer cell invasion and migration in vitro and in vivo. Furthermore, metabolomic analysis showed some important regulated metabolomic pathways such as amino acids-related pathways. Many amino acids were downregulated in Setd7 KO and CPH mice. Future investigations should be done to better understand the mechanism of action of CPH and Setd7 at the molecular level in regulating the different signaling pathways of lung cancer. Additionally, further in vivo investigations, such as different lung cancer models and dose–response studies, should be evaluated.

## Supplementary Information

Below is the link to the electronic supplementary material.

Supplementary file1 (DOCX 15 KB)

Supplementary file2 (PPTX 85 KB)

## Data Availability

No datasets were generated or analysed during the current study.

## References

[1] Kratzer TB et al (2024) Lung cancer statistics, 2023. Cancer. 10.1002/cncr.35128 10.1002/cncr.35128 38279776

[2] Tamura T et al (2015) Specific organ metastases and survival in metastatic non-small-cell lung cancer. Mol Clin Oncol 3(1):217–221. 10.3892/mco.2014.410 25469298 10.3892/mco.2014.410 PMC4251107

[3] Cheng Y, Zhang T, Xu Q (2021) Therapeutic advances in non-small cell lung cancer: focus on clinical development of targeted therapy and immunotherapy. MedComm (2020) 2(4):692–729. 10.1002/mco2.105 34977873 10.1002/mco2.105 PMC8706764

[4] Dolgin E (2010) Behind the paper: muzzling muscle spasticity. Nat Med 16(6):637–637. 10.1038/nm.2169 20512125 10.1038/nm.2169

[5] Noble RE (1969) Effect of cyproheptadine on appetite and weight gain in adults. J Am Med Assoc 209(13):2054–3000. 10.1001/jama.209.13.2054 4897366

[6] Feng YM et al (2015) Cyproheptadine, an antihistaminic drug, inhibits proliferation of hepatocellular carcinoma cells by blocking cell cycle progression through the activation of P38 MAP kinase. BMC Cancer. 10.1186/s12885-015-1137-9 10.1186/s12885-015-1137-9 PMC4383201 25886177

[7] Li J et al (2013) Cyproheptadine-induced myeloma cell apoptosis is associated with inhibition of the PI3K/AKT signaling. Eur J Haematol 91(6):514–521. 10.1111/ejh.12193 24033664 10.1111/ejh.12193

[8] Mao XL et al (2008) Cyproheptadine displays preclinical activity in myeloma and leukemia. Blood 112(3):760–769. 10.1182/blood-2008-02-142687 18502826 10.1182/blood-2008-02-142687

[9] Paoluzzi L et al (2009) The anti-histaminic cyproheptadine synergizes the antineoplastic activity of bortezomib in mantle cell lymphoma through its effects as a histone deacetylase inhibitor. Br J Haematol 146(6):656–659. 10.1111/j.1365-2141.2009.07797.x 19604235 10.1111/j.1365-2141.2009.07797.x

[10] Takemoto Y et al (2016) Identification of cyproheptadine as an inhibitor of SET domain containing lysine methyltransferase 7/9 (Set7/9) that regulates estrogen-dependent transcription. J Med Chem 59(8):3650–3660. 10.1021/acs.jmedchem.5b01732 27088648 10.1021/acs.jmedchem.5b01732

[11] Hsieh HY et al (2016) Cyproheptadine exhibits antitumor activity in urothelial carcinoma cells by targeting GSK3beta to suppress mTOR and beta-catenin signaling pathways. Cancer Lett 370(1):56–65. 10.1016/j.canlet.2015.09.018 26454215 10.1016/j.canlet.2015.09.018

[12] Jou YC et al (2021) Cyproheptadine, an epigenetic modifier, exhibits anti-tumor activity by reversing the epigenetic silencing of IRF6 in urothelial carcinoma. Cancer Cell Int. 10.1186/s12935-021-01925-9 10.1186/s12935-021-01925-9 PMC8054409 33874979

[13] Feng YM et al (2012) Unexpected remission of hepatocellular carcinoma (HCC) with lung metastasis to the combination therapy of thalidomide and cyproheptadine: report of two cases and a preliminary HCC cell line study. BMJ Case Rep. 10.1136/bcr-2012-007180 10.1136/bcr-2012-007180 PMC4543862 23076705

[14] Feng YM et al (2021) Efficacy of cyproheptadine monotherapy in hepatocellular carcinoma with bone metastasis: a case report. Front Oncol. 10.3389/fonc.2021.620212 10.3389/fonc.2021.620212 PMC8563693 34745929

[15] Feng YM et al (2015) Cyproheptadine significantly improves the overall and progression-free survival of sorafenib-treated advanced HCC patients. Jpn J Clin Oncol 45(4):336–342. 10.1093/jjco/hyv007 25646358 10.1093/jjco/hyv007 PMC4376992

[16] Monteiro FL, Williams C, Helguero LA (2022) A systematic review to define the multi-faceted role of lysine methyltransferase SETD7 in cancer. Cancers (Basel). 10.3390/cancers14061414 10.3390/cancers14061414 PMC8946661 35326563

[17] Shen C et al (2015) SET7/9 regulates cancer cell proliferation by influencing beta-catenin stability. FASEB J 29(10):4313–4323. 10.1096/fj.15-273540 26116705 10.1096/fj.15-273540

[18] Chen YY et al (2016) Increased expression of SETD7 promotes cell proliferation by regulating cell cycle and indicates poor prognosis in hepatocellular carcinoma. PLoS ONE. 10.1371/journal.pone.0154939 10.1371/journal.pone.0154939 PMC4868314 27183310

[19] Wang C et al (2018) Histone methyltransferase Setd7 regulates Nrf2 signaling pathway by phenethyl isothiocyanate and ursolic acid in human prostate cancer cells. Mol Nutr Food Res. 10.1002/mnfr.201700840 10.1002/mnfr.201700840 PMC6226019 29383876

[20] Wang C et al (2018) Transcriptomic analysis of histone methyltransferase Setd7 knockdown and phenethyl isothiocyanate in human prostate cancer cells. J Anticancer Res 38(11):6069–6083. 10.21873/anticanres.12957 10.21873/anticanres.12957 30396921

[21] Monteiro FL, Williams C, Helguero LA (2022) A systematic review to define the multi-faceted role of lysine methyltransferase SETD7 in cancer. Cancers. 10.3390/cancers14061414 10.3390/cancers14061414 PMC8946661 35326563

[22] Daks A et al (2021) Set7/9 controls proliferation and genotoxic drug resistance of NSCLC cells. Biochem Biophys Res Commun 572:41–48. 10.1016/j.bbrc.2021.07.086 34343833 10.1016/j.bbrc.2021.07.086

[23] Lezina L et al (2015) KMT Set7/9 affects genotoxic stress response via the Mdm2 axis. Oncotarget 6(28):25843–25855. 10.18632/oncotarget.4584 26317544 10.18632/oncotarget.4584 PMC4694870

[24] Lezina L et al (2014) KMTase Set7/9 is a critical regulator of E2F1 activity upon genotoxic stress. Cell Death Differ 21(12):1889–1899. 10.1038/cdd.2014.108 25124555 10.1038/cdd.2014.108 PMC4227146

[25] Kontaki H, Talianidis I (2010) Lysine methylation regulates E2F1-induced cell death. Mol Cell 39(1):152–160. 10.1016/j.molcel.2010.06.006 20603083 10.1016/j.molcel.2010.06.006

[26] de Albuquerque Almeida Batista I, Helguero LA (2018) Biological processes and signal transduction pathways regulated by the protein methyltransferase SETD7 and their significance in cancer. Signal Transduct Target Ther 3(1):19. 10.1038/s41392-018-0017-6 10.1038/s41392-018-0017-6 PMC6043541 30013796

[27] Lehnertz B et al (2011) p53-dependent transcription and tumor suppression are not affected in Set7/9-deficient mice. Mol Cell 43(4):673–680. 10.1016/j.molcel.2011.08.006 21855805 10.1016/j.molcel.2011.08.006

[28] Sarwar MS et al (2023) The environmental carcinogen benzo[a]pyrene regulates epigenetic reprogramming and metabolic rewiring in a two-stage mouse skin carcinogenesis model. Carcinogenesis 44(5):436–449. 10.1093/carcin/bgad024 37100755 10.1093/carcin/bgad024 PMC10414144

[29] Sarwar MS et al (2023) Metabolic rewiring and epigenetic reprogramming in leptin receptor-deficient db/db diabetic nephropathy mice. Eur J Pharmacol 953:175866. 10.1016/j.ejphar.2023.175866 37331680 10.1016/j.ejphar.2023.175866

[30] Sarwar MS et al (2024) Triterpenoid ursolic acid regulates the environmental carcinogen benzo[a]pyrene-driven epigenetic and metabolic alterations in SKH-1 hairless mice for skin cancer interception. Carcinogenesis 45(5):288–299. 10.1093/carcin/bgae009 38466106 10.1093/carcin/bgae009 PMC11102768

[31] Si WZ et al (2020) SET7/9 promotes multiple malignant processes in breast cancer development via RUNX2 activation and is negatively regulated by TRIM21. Cell Death Dis. 10.1038/s41419-020-2350-2 10.1038/s41419-020-2350-2 PMC7044199 32102992

[32] Fu L et al (2016) Set7 mediated Gli3 methylation plays a positive role in the activation of Sonic Hedgehog pathway in mammals. Elife. 10.7554/eLife.15690 10.7554/eLife.15690 PMC4884081 27146893

[33] Kim Y et al (2016) Methylation-dependent regulation of HIF-1α stability restricts retinal and tumour angiogenesis. Nat Commun 7(1):10347. 10.1038/ncomms10347 26757928 10.1038/ncomms10347 PMC4735525

[34] Chiang CY et al (2023) Methylation of KRAS by SETD7 promotes KRAS degradation in non-small cell lung cancer. Cell Rep 42(9):113003. 10.1016/j.celrep.2023.113003 37682707 10.1016/j.celrep.2023.113003

[35] Fujiwara T et al (2016) Steric structure-activity relationship of cyproheptadine derivatives as inhibitors of histone methyltransferase Set7/9. Bioorg Med Chem 24(18):4318–4323. 10.1016/j.bmc.2016.07.024 27448773 10.1016/j.bmc.2016.07.024

[36] Hirano T et al (2018) Development of novel inhibitors for histone methyltransferase SET7/9 based on cyproheptadine. ChemMedChem 13(15):1530–1540. 10.1002/cmdc.201800233 29882380 10.1002/cmdc.201800233

[37] Rohde JM et al (2018) Discovery and optimization of piperazine-1-thiourea-based human phosphoglycerate dehydrogenase inhibitors. Bioorg Med Chem 26(8):1727–1739. 10.1016/j.bmc.2018.02.016 29555419 10.1016/j.bmc.2018.02.016 PMC5891386

[38] Feng YM et al (2015) Cyproheptadine, an antihistaminic drug, inhibits proliferation of hepatocellular carcinoma cells by blocking cell cycle progression through the activation of P38 MAP kinase. BMC Cancer 15:134. 10.1186/s12885-015-1137-9 25886177 10.1186/s12885-015-1137-9 PMC4383201

[39] Jou YC et al (2021) Cyproheptadine, an epigenetic modifier, exhibits anti-tumor activity by reversing the epigenetic silencing of IRF6 in urothelial carcinoma. Cancer Cell Int 21(1):226. 10.1186/s12935-021-01925-9 33874979 10.1186/s12935-021-01925-9 PMC8054409

[40] Castaneda M et al (2022) Mechanisms of cancer metastasis. Semin Cancer Biol 87:17–31. 10.1016/j.semcancer.2022.10.006 36354098 10.1016/j.semcancer.2022.10.006

[41] Liu R et al (2023) Methylation across the central dogma in health and diseases: new therapeutic strategies. Signal Transduct Target Ther 8(1):310. 10.1038/s41392-023-01528-y 37620312 10.1038/s41392-023-01528-y PMC10449936

[42] Karno B, Edwards DN, Chen J (2023) Metabolic control of cancer metastasis: role of amino acids at secondary organ sites. Oncogene 42(47):3447–3456. 10.1038/s41388-023-02868-3 37848626 10.1038/s41388-023-02868-3 PMC11323979

[43] Rinaldi G et al (2021) In vivo evidence for serine biosynthesis-defined sensitivity of lung metastasis, but not of primary breast tumors, to mTORC1 inhibition. Mol Cell 81(2):386-397.e7. 10.1016/j.molcel.2020.11.027 33340488 10.1016/j.molcel.2020.11.027 PMC9161668

[44] Kiweler N et al (2022) Mitochondria preserve an autarkic one-carbon cycle to confer growth-independent cancer cell migration and metastasis. Nat Commun 13(1):2699. 10.1038/s41467-022-30363-y 35577770 10.1038/s41467-022-30363-y PMC9110368

[45] Yao S et al (2022) Phase 1 trial of ADI-PEG 20 and liposomal doxorubicin in patients with metastatic solid tumors. Cancer Med 11(2):340–347. 10.1002/cam4.4446 34841717 10.1002/cam4.4446 PMC8729058

[46] Qiu F et al (2014) Arginine starvation impairs mitochondrial respiratory function in ASS1-deficient breast cancer cells. Sci Signal. 10.1126/scisignal.2004761 10.1126/scisignal.2004761 PMC4229039 24692592

[47] Stelter L et al (2013) Evaluation of arginine deiminase treatment in melanoma xenografts using (18)F-FLT PET. Mol Imaging Biol 15(6):768–775. 10.1007/s11307-013-0655-6 23722880 10.1007/s11307-013-0655-6 PMC4537614

