# Supplementary material for: Cyproheptadine inhibits in vitro and in vivo lung metastasis and drives metabolic rewiring
Source: Mol Biol Rep. 2024 Nov 10;51(1):1139. doi: 10.1007/s11033-024-10033-6 (PMC11551078; doi:10.1007/s11033-024-10033-6)
Supplement: Supplementary file 1 — Supplementary file1 (DOCX 15 KB) [file 11033_2024_10033_MOESM1_ESM.docx]

**Supplemental Material**

**Table S1 RT-PCR primers**

| **RT-PCR Primers** | **Forward (5’ – 3’)** | **Reverse (5’ – 3’)** |
| --- | --- | --- |
| *YAP* | CCAGACGACTTCCTCAACAGTG | GCATCTCCTTCCAGTGTGCCAA |
| MDM2 | GATTGCCTGGATCAGGATTCAGTT | GGCTGTAATCTTCCGAGTCCAGG |
| P53 | TGAAACGCCGACCTATCCTTA | GGCACAAACACGAACCTCAAA |
| Mmp1a | CCTTGATGAGACGTGGACCAA | ATGTGGTGTTGTTGCACCTGT |
| *Mmp3* | GGCCTGGAACAGTCTTGGC | TGTCCATCGTTCATCATCGTCA |
| *Mmp13* | TGTTTGCAGAGCACTACTTGAA | CAGTCACCTCTAAGCCAAAGAAA |
| *Act* | ATGAACGACGTAGCCATTGTG | TTGTAGCCAATAAAGGTGCCAT |
| *Cdhr* | CAGTTCCGAGGTCTACACCTT | TGAATCGGGAGTCTTCCGAAAA |
| *Mtor* | CAGTTCGCCAGTGGACTGAAG | GCTGGTCATAGAAGCGAGTAGAC |
| *Vcam* | TTGGGAGCCTCAACGGTACT | GCAATCGTTTTGTATTCAGGGGA |
| *Vim1* | CGTCCACACGCACCTACAG | GGGGGATGAGGAATAGAGGCT |
| *Vegfd* | AACAGATCCGAGCAGCTTCTA | TTTTGAGCTTCAACCGGCATC |
| *GAPDH* | CATCACTGCCACCCAGAAGACTG | ATGCCAGTGAGCTTCCCGTTCAG |
