# Supplementary figures and images for: Cyproheptadine inhibits in vitro and in vivo lung metastasis and drives metabolic rewiring
Source: Mol Biol Rep. 2024 Nov 10;51(1):1139. doi: 10.1007/s11033-024-10033-6 (PMC11551078; doi:10.1007/s11033-024-10033-6)

## Slide 1
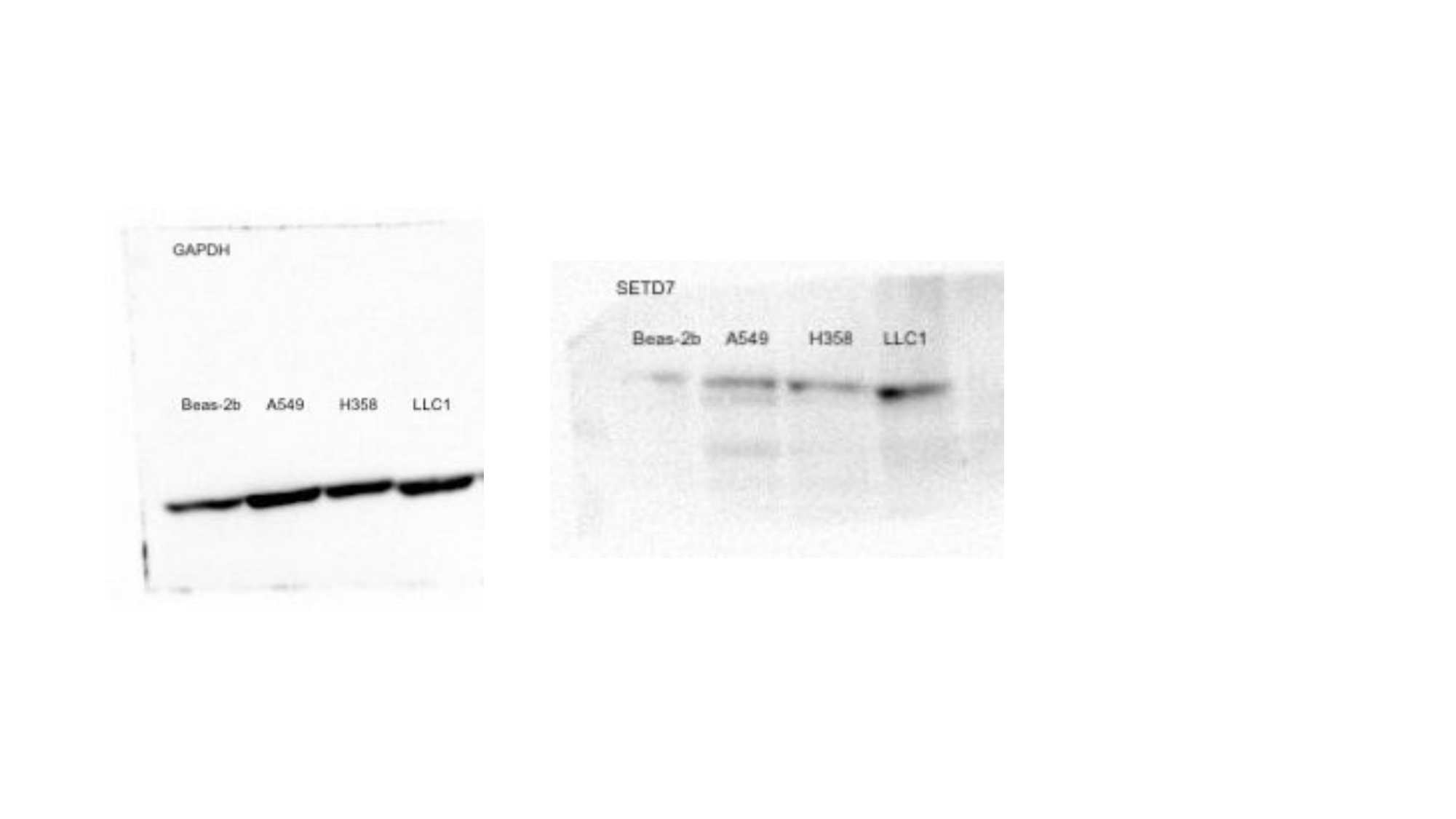

Supplement: Supplementary file 2 — Supplementary file2 (PPTX 85 KB) [file 11033_2024_10033_MOESM2_ESM.pptx]
